# Crystal structure of *N*-(4-hy­droxy­benz­yl)acetone thio­semicarbazone

**DOI:** 10.1107/S2056989017012129

**Published:** 2017-08-25

**Authors:** Saray Argibay-Otero, Ezequiel M. Vázquez-López

**Affiliations:** a Departamento de Química Inorgánica, Facultade de Química, Instituto de Investigación Sanitaria Galicia Sur – Universidade de Vigo, Campus Universitario, E-36310 Vigo, Galicia, Spain

**Keywords:** crystal structure, thio­semicarbazone, thio­urea, hydrogen bonding

## Abstract

The structure of the title compound, C_11_H_15_N_3_OS, shows the flexibility due to the methyl­ene group at the thio­amide N atom in the side chain, resulting in the mol­ecule being non-planar. The dihedral angle between the plane of the benzene ring and that defined by the atoms of the thio­semicarbazide arm is 79.847 (4)°. In the crystal, the donor–acceptor hydrogen-bond character of the –OH group dominates the inter­molecular associations, acting as a donor in an O—H⋯S hydrogen bond, as well as being a double acceptor in a centrosymmetric cyclic bridging N—H⋯O,O′ inter­action [graph set *R*_2_^2^(4)]. The result is a one-dimensional duplex chain structure, extending along [111]. The usual N—H⋯S hydrogen-bonding association common in thio­semicarbazone crystal structures is not observed.

## Abstract 2

The inclusion of a methyl­ene group at the thio­amidic N atom of the acetone thio­semicarbazone derivative endows the mol­ecule with greater flexibility and different pathways of association compared to those usually observed in the crystalline structures of these compounds.

## Chemical context   

**Figure 1:**
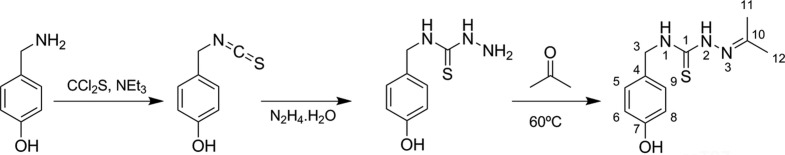
Reaction scheme for the synthesis of acTSC.

Thio­semicarbazones (TSCs) are an inter­esting group of compounds because they show diverse biological properties (Serda *et al.*, 2012 ▸) and pharmacological activities (Lukmantara *et al.*, 2013 ▸). They can be easily functionalized to yield different supra­molecular arrays through inter­molecular hydrogen-bonding inter­actions (Nuñez-Montenegro *et al.*, 2017 ▸), by selection of suitable aldehyde or ketone reagents. In addition, metal coordination may be used to orient some of their substituents to optimize the inter­action with biomolecules (*e.g.* see Nuñez-Montenegro *et al.*, 2014 ▸). In the present paper, we describe the synthesis and crystal structure of a TSC derivative (Figs. 1 ▸), namely *N*-(4-hy­droxy­benz­yl)acetone thio­semicarbazone (acTSC), having a 4-hy­droxy­benzyl substituent at the thio­amide N atom (N1), in which the –CH_2_– group provides more flexibility to establish inter­molecular associations.
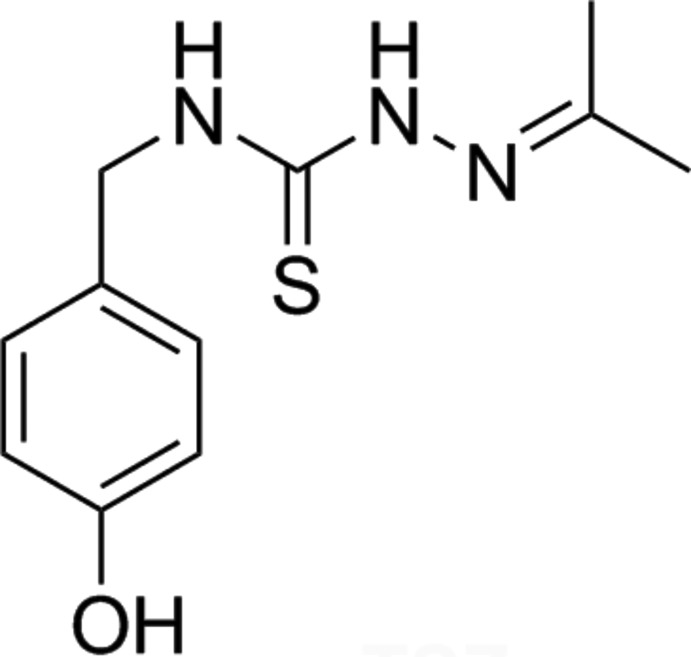


## Structural commentary   

**Figure 2:**
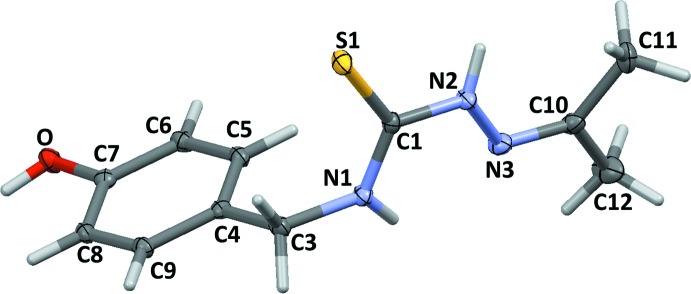
The mol­ecular structure of acTSC, with displacement ellipsoids drawn at the 40% probability level.

In the acTSC mol­ecule (Fig. 2 ▸), the bond lengths (S1=C1 and C10=N3) and angles in the thio­semicarbazide arm are similar to those observed in other thio­semicarbazones, suggesting that the thione form is predominant. This arm is almost planar, probably due to some π-delocation (r.m.s. deviation of 0.0516 Å for the plane defined by atoms S1/C1/N1/N2/N3). Nevertheless, the ethyl­ene group at N1 allows an almost orthogonal orientation relative to the phenolic substituent group, with a dihedral angle between the two planes of 79.847 (4)°. The interatomic distance N1⋯N3 inter­action [2.6074 (18) Å] suggests some kind of intramolecular interaction.

## Supra­molecular features   

**Figure 3:**
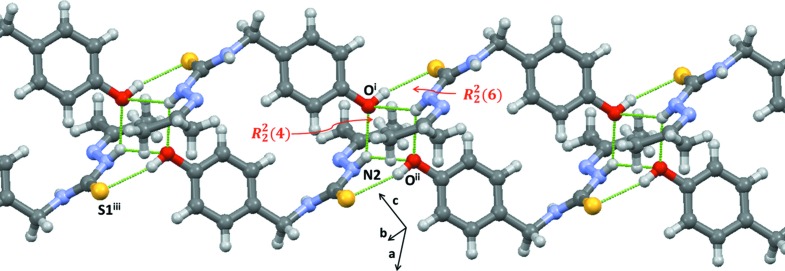
Inter­molecular hydrogen-bonding associations between mol­ecules in the crystal structure of acTSC, shown as dashed lines.

The association of the mol­ecules is strongly affected by the donor–acceptor character of the –OH group, while the usual N—H⋯S hydrogen bonds observed in most TSC structures (Nuñez-Montenegro *et al.*, 2017 ▸; Pino-Cuevas *et al.*, 2014 ▸) are absent. The phenolic –OH group forms an inter­molecular hydrogen bond with a S-atom acceptor (O—H0⋯S1^iii^; Table 1 ▸), while the N2—H group establishes two different hydrogen-bonding inter­actions with different phenolic O-atom acceptors. The shortest of these is N2—H2⋯O^i^ (Table 1 ▸), which generates a centrosymmetric cyclic 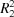
(4) ring-motif association (Etter, 1990 ▸) and also forms a conjoined cyclic 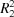
(6) association *via* an O—H⋯S inter­action (see Fig. 3 ▸). The second of the three-centre hydrogen-bonding inter­actions (N2—H2⋯O^ii^) extends the structure into one-dimensional duplex chains along [111] (Fig. 3 ▸).

## Database survey   

For related structures of thio­semicarbazones derived from acetone, see: Yamin *et al.* (2014 ▸); Basu & Das (2011 ▸); Venkatraman *et al.* (2005 ▸); Jian *et al.* (2005 ▸). For the metal-coordination properties of thio­semicarbazones, see: Paterson & Donnelly (2011 ▸); Casas *et al.* (2000 ▸). For acetone derivatives, see, for example, Su *et al.* (2013 ▸); Nuñez-Montenegro *et al.* (2014 ▸); Swesi *et al.* (2006 ▸); Paek *et al.* (1997 ▸).

## Synthesis and crystallization   

The reaction scheme for the synthesis of the title compound is shown in Fig. 1 ▸. The primary amine 4-hy­droxy­benzyl­amine was converted to the corresponding iso­thio­cyanate by reaction with thio­phosgene (Sharma, 1978 ▸). This iso­thio­cyanate was treated with hydrazide to form the thio­semicarbazide, as described previously (Reis *et al.*, 2011 ▸). Finally, this compound was reacted with acetone in order to synthesize the desired thio­semicarbazone. In a typical synthesis, 3.4 g (0.017 mol) of thio­semicarbazide was dissolved in acetone (20 ml) and heated to 60°C for 20 min (Fig. 1 ▸). This solution was concentrated and the resultant residue was purified using a silica column (AcOEt–hexane 30%). This solution was vacuum dried giving 1.96 g of acTSC. The solution was also used to obtain single crystals by slow evaporation (yield 48%; m.p. 165°C). C_11_H_15_N_3_OS requires: C 55.7, H 6.4, N 17.7%; found C 55.8, H 7.1,N 16.9%. MS–ESI [*m*/*z* (%)]: 238 (100) [*M* + H]^+^. IR (ATR, ν/cm^−1^): 3241 (*b*) ν(NH, OH); 1536 (*w*), 1508 (*s*) ν(C=N); 784 (*w*) ν(C=S). ^1^H NMR (DMSO-*d*_6_): 9.95 (*s*, 1H, N2H), 9.26 (*s*, 1H, OH), 8.46 (*t*, ^3^ *J*_H-NH_ = 6.2Hz, 1H, N1H), 7.15 (*d*, ^3^ *J*_H-H_ = 8.5Hz, 2H, C5H, C9H), 6.70 (*d*, ^3^ *J*_H-H_ = 8.5Hz, 2H, C6H, C8H), 4.65 (*d*, ^3^ *J*_H-H_ = 6.2Hz, 2H, C3H), 1.92 (*d*, ^3^ *J*_H-H_ = 8.5Hz, 6H, C11H, C12H).

## Refinement   

Crystal data, data collection and structure refinement details are summarized in Table 2 ▸. Inter­active H atoms on O and N atoms were located in difference Fourier analyses and were allowed to freely refine, with *U*_iso_(H) = 1.2*U*_eq_(O,N) and riding. Other H atoms were included at calculated sites and allowed to ride, with *U*_iso_(H) = 1.2*U*_eq_(aromatic and methyl­ene C) or 1.5*U*_eq_(methyl C).

## Supplementary Material

Crystal structure: contains datablock(s) I, global. DOI: 10.1107/S2056989017012129/zs2385sup1.cif

Structure factors: contains datablock(s) I. DOI: 10.1107/S2056989017012129/zs2385Isup2.hkl

CCDC reference: 1570200

Additional supporting information:  crystallographic information; 3D view; checkCIF report

## Figures and Tables

**Table 1:** Hydrogen-bond geometry (Å, °)

*D*—H⋯*A*	*D*—H	H⋯*A*	*D*⋯*A*	*D*—H⋯*A*
N2—H2⋯O^i^	0.848 (17)	2.292 (17)	2.9955 (15)	140.6 (14)
N2—H2⋯O^ii^	0.848 (17)	2.434 (16)	3.1333 (15)	140.3 (14)
O—H0⋯S1^iii^	0.857 (19)	2.299 (19)	3.1349 (10)	165.2 (16)

Symmetry codes: (i) 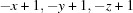; (ii) 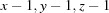; (iii) 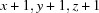.

**Table 2:** Experimental details

Crystal data	
Chemical formula	C_11_H_15_N_3_OS
*M*_r_	237.32
Crystal system, space group	Triclinic, *P* 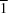
Temperature (K)	100
*a*, *b*, *c* (Å)	8.2799 (8), 8.9169 (9), 9.7451 (10)
α, β, γ (°)	104.597 (3), 112.569 (3), 105.220 (3)
*V* (Å^3^)	588.7 (1)
*Z*	2
Radiation type	Mo *K*α
μ (mm^−1^)	0.26
Crystal size (mm)	0.18 × 0.11 × 0.11

Data collection	
Diffractometer	Bruker D8 Venture Photon 100 CMOS
Absorption correction	Multi-scan (*SADABS*; Bruker, 2014 ▸)
*T*_min_, *T*_max_	0.638, 0.746
No. of measured, independent and observed [*I* > 2σ(*I*)] reflections	17083, 2911, 2562
*R*_int_	0.043
(sin θ/λ)_max_ (Å^−1^)	0.667

Refinement	
*R*[*F* ^2^ > 2σ(*F* ^2^)], *wR*(*F* ^2^), *S*	0.032, 0.082, 1.00
No. of reflections	2911
No. of parameters	156
H-atom treatment	H atoms treated by a mixture of independent and constrained refinement
Δρ_max_, Δρ_min_ (e Å^−3^)	0.29, −0.28

Computer programs: *APEX3* (Bruker, 2014 ▸), *SAINT* (Bruker, 2014 ▸), *SHELXS2013* (Sheldrick, 2015*a* ▸), *SHELXL2013* (Sheldrick, 2015*b* ▸) and *Mercury* (Bruno *et al.*, 2002 ▸).

## supplementary crystallographic information

### Crystal data

**Table:**

C_11_H_15_N_3_OS	*F*(000) = 252
*M**_r_* = 237.32	*D*_x_ = 1.339 Mg m^−^^3^
Triclinic, *P*1	Melting point: 438 K
*a* = 8.2799 (8) Å	Mo *K*α radiation, λ = 0.71073 Å
*b* = 8.9169 (9) Å	Cell parameters from 9917 reflections
*c* = 9.7451 (10) Å	θ = 2.5–28.3°
α = 104.597 (3)°	µ = 0.26 mm^−^^1^
β = 112.569 (3)°	*T* = 100 K
γ = 105.220 (3)°	Prism, colourless
*V* = 588.7 (1) Å^3^	0.18 × 0.11 × 0.11 mm
*Z* = 2	

### Data collection

**Table:**

Bruker D8 Venture Photon 100 CMOS diffractometer	2562 reflections with *I* > 2σ(*I*)
φ and ω scans	*R*_int_ = 0.043
Absorption correction: multi-scan (SADABS; Bruker, 2014)	θ_max_ = 28.3°, θ_min_ = 2.5°
*T*_min_ = 0.638, *T*_max_ = 0.746	*h* = −11→11
17083 measured reflections	*k* = −11→11
2911 independent reflections	*l* = −12→13

### Refinement

**Table:**

Refinement on *F*^2^	0 restraints
Least-squares matrix: full	Hydrogen site location: mixed
*R*[*F*^2^ > 2σ(*F*^2^)] = 0.032	H atoms treated by a mixture of independent and constrained refinement
*wR*(*F*^2^) = 0.082	*w* = 1/[σ^2^(*F*_o_^2^) + (0.0325*P*)^2^ + 0.3538*P*] where *P* = (*F*_o_^2^ + 2*F*_c_^2^)/3
*S* = 1.00	(Δ/σ)_max_ < 0.001
2911 reflections	Δρ_max_ = 0.29 e Å^−^^3^
156 parameters	Δρ_min_ = −0.28 e Å^−^^3^

### Special details

**Table:**

Geometry. All esds (except the esd in the dihedral angle between two l.s. planes) are estimated using the full covariance matrix. The cell esds are taken into account individually in the estimation of esds in distances, angles and torsion angles; correlations between esds in cell parameters are only used when they are defined by crystal symmetry. An approximate (isotropic) treatment of cell esds is used for estimating esds involving l.s. planes.

### Fractional atomic coordinates and isotropic or equivalent isotropic displacement parameters (Å^2^)

**Table:**

	*x*	*y*	*z*	*U*_iso_*/*U*_eq_	
C12	0.5032 (2)	−0.1953 (2)	0.3563 (2)	0.0345 (4)	
H12A	0.6301	−0.1042	0.4309	0.052*	
H12B	0.5165	−0.2971	0.3036	0.052*	
H12C	0.4356	−0.2199	0.4166	0.052*	
C11	0.1912 (2)	−0.26338 (16)	0.10998 (17)	0.0243 (3)	
H11A	0.1070	−0.2506	0.1562	0.036*	
H11B	0.1842	−0.3793	0.0828	0.036*	
H11C	0.1508	−0.2401	0.0118	0.036*	
S1	0.33739 (4)	0.28903 (4)	0.00401 (4)	0.02014 (10)	
O	0.96910 (14)	0.95385 (12)	0.83590 (11)	0.0206 (2)	
H0	1.063 (3)	1.046 (2)	0.864 (2)	0.031*	
N2	0.37419 (16)	0.05734 (13)	0.12372 (13)	0.0170 (2)	
H2	0.254 (2)	0.024 (2)	0.0853 (19)	0.020*	
N1	0.65479 (15)	0.27673 (13)	0.21157 (13)	0.0163 (2)	
H1	0.701 (2)	0.225 (2)	0.2663 (19)	0.020*	
N3	0.47907 (15)	0.00499 (14)	0.23774 (13)	0.0194 (2)	
C6	0.79084 (18)	0.67223 (16)	0.64146 (15)	0.0173 (2)	
H6	0.7308	0.6484	0.7038	0.021*	
C3	0.77639 (18)	0.44581 (15)	0.24342 (15)	0.0171 (2)	
H3A	0.7100	0.4789	0.1551	0.021*	
H3B	0.8951	0.4438	0.2427	0.021*	
C1	0.46524 (17)	0.20611 (15)	0.12097 (14)	0.0147 (2)	
C4	0.82883 (17)	0.57817 (15)	0.40388 (14)	0.0148 (2)	
C7	0.92864 (17)	0.83296 (15)	0.69457 (14)	0.0158 (2)	
C9	0.96926 (17)	0.73918 (15)	0.46107 (15)	0.0170 (2)	
H9	1.0319	0.7622	0.4003	0.020*	
C8	1.01956 (17)	0.86683 (15)	0.60508 (15)	0.0164 (2)	
H8	1.1150	0.9760	0.6419	0.020*	
C5	0.74164 (17)	0.54680 (15)	0.49646 (15)	0.0162 (2)	
H5	0.6466	0.4376	0.4600	0.019*	
C10	0.39253 (19)	−0.14134 (17)	0.23078 (16)	0.0197 (3)	

### Atomic displacement parameters (Å^2^)

**Table:**

	*U*^11^	*U*^22^	*U*^33^	*U*^12^	*U*^13^	*U*^23^
C12	0.0274 (8)	0.0421 (9)	0.0502 (10)	0.0188 (7)	0.0187 (7)	0.0374 (8)
C11	0.0297 (7)	0.0153 (6)	0.0263 (7)	0.0065 (5)	0.0131 (6)	0.0090 (5)
S1	0.01646 (16)	0.01708 (15)	0.02248 (17)	0.00497 (12)	0.00379 (13)	0.01202 (12)
O	0.0182 (5)	0.0184 (4)	0.0173 (4)	0.0022 (4)	0.0071 (4)	0.0030 (4)
N2	0.0138 (5)	0.0164 (5)	0.0197 (5)	0.0056 (4)	0.0053 (4)	0.0104 (4)
N1	0.0152 (5)	0.0145 (5)	0.0182 (5)	0.0059 (4)	0.0059 (4)	0.0083 (4)
N3	0.0174 (5)	0.0228 (5)	0.0238 (6)	0.0108 (4)	0.0097 (5)	0.0157 (5)
C6	0.0154 (6)	0.0201 (6)	0.0178 (6)	0.0059 (5)	0.0085 (5)	0.0101 (5)
C3	0.0151 (6)	0.0164 (6)	0.0187 (6)	0.0042 (5)	0.0081 (5)	0.0076 (5)
C1	0.0163 (6)	0.0135 (5)	0.0139 (5)	0.0060 (5)	0.0075 (5)	0.0046 (4)
C4	0.0131 (5)	0.0158 (5)	0.0161 (6)	0.0072 (5)	0.0055 (5)	0.0082 (5)
C7	0.0134 (5)	0.0169 (6)	0.0144 (6)	0.0067 (5)	0.0036 (5)	0.0065 (4)
C9	0.0154 (6)	0.0182 (6)	0.0202 (6)	0.0065 (5)	0.0094 (5)	0.0110 (5)
C8	0.0131 (5)	0.0146 (5)	0.0194 (6)	0.0041 (4)	0.0056 (5)	0.0086 (5)
C5	0.0131 (5)	0.0154 (5)	0.0186 (6)	0.0042 (4)	0.0060 (5)	0.0087 (5)
C10	0.0213 (6)	0.0225 (6)	0.0263 (7)	0.0131 (5)	0.0155 (5)	0.0152 (5)

### Geometric parameters (Å, º)

**Table:**

S1—C1	1.6959 (14)	C8—C9	1.3914 (18)
O—C7	1.3708 (16)	C10—C11	1.498 (2)
O—H0	0.86 (2)	C10—C12	1.495 (2)
N1—C3	1.4527 (19)	C3—H3A	0.9900
N1—C1	1.3328 (19)	C3—H3B	0.9900
N2—N3	1.3929 (17)	C5—H5	0.9500
N2—C1	1.3554 (19)	C6—H6	0.9500
N3—C10	1.284 (2)	C8—H8	0.9500
N1—H1	0.839 (18)	C9—H9	0.9500
N2—H2	0.849 (18)	C11—H11A	0.9800
C3—C4	1.5186 (18)	C11—H11B	0.9800
C4—C5	1.391 (2)	C11—H11C	0.9800
C4—C9	1.395 (2)	C12—H12A	0.9800
C5—C6	1.3914 (19)	C12—H12B	0.9800
C6—C7	1.392 (2)	C12—H12C	0.9800
C7—C8	1.391 (2)		
			
C7—O—H0	111.1 (13)	N1—C3—H3B	109.00
C1—N1—C3	124.73 (12)	C4—C3—H3A	109.00
N2—N3—C10	116.82 (12)	C4—C3—H3B	109.00
S1—C1—N1	124.19 (11)	H3A—C3—H3B	108.00
S1—C1—N2	119.75 (11)	C4—C5—H5	119.00
C1—N1—H1	115.1 (12)	C6—C5—H5	119.00
C3—N1—H1	119.1 (12)	C5—C6—H6	120.00
N1—C1—N2	116.05 (12)	C7—C6—H6	120.00
N3—N2—H2	120.9 (12)	C7—C8—H8	120.00
C1—N2—H2	116.2 (13)	C9—C8—H8	120.00
N1—C3—C4	113.39 (12)	C4—C9—H9	119.00
C3—C4—C5	122.91 (12)	C8—C9—H9	119.00
C5—C4—C9	118.17 (12)	C10—C11—H11A	109.00
C3—C4—C9	118.92 (12)	C10—C11—H11B	109.00
C4—C5—C6	121.31 (13)	C10—C11—H11C	109.00
C5—C6—C7	119.54 (13)	H11A—C11—H11B	109.00
O—C7—C6	117.57 (13)	H11A—C11—H11C	109.00
C6—C7—C8	120.21 (12)	H11B—C11—H11C	109.00
O—C7—C8	122.21 (12)	C10—C12—H12A	109.00
C7—C8—C9	119.30 (13)	C10—C12—H12B	109.00
C4—C9—C8	121.46 (13)	C10—C12—H12C	109.00
N3—C10—C12	116.82 (14)	H12A—C12—H12B	109.00
C11—C10—C12	116.61 (14)	H12A—C12—H12C	109.00
N3—C10—C11	126.57 (13)	H12B—C12—H12C	109.00
N1—C3—H3A	109.00		
			
C3—N1—C1—S1	10.03 (19)	C3—C4—C9—C8	178.25 (13)
C3—N1—C1—N2	−171.02 (12)	C3—C4—C5—C6	−178.75 (14)
C1—N1—C3—C4	97.14 (15)	C9—C4—C5—C6	0.6 (2)
C1—N2—N3—C10	−175.62 (14)	C5—C4—C9—C8	−1.1 (2)
N3—N2—C1—S1	−170.98 (10)	C4—C5—C6—C7	0.7 (2)
N3—N2—C1—N1	10.02 (19)	C5—C6—C7—C8	−1.4 (2)
N2—N3—C10—C12	−178.18 (13)	C5—C6—C7—O	178.34 (13)
N2—N3—C10—C11	1.5 (2)	O—C7—C8—C9	−178.83 (13)
N1—C3—C4—C9	169.60 (13)	C6—C7—C8—C9	0.9 (2)
N1—C3—C4—C5	−11.0 (2)	C7—C8—C9—C4	0.4 (2)

### Hydrogen-bond geometry (Å, º)

**Table:**

*D*—H···*A*	*D*—H	H···*A*	*D*···*A*	*D*—H···*A*
N2—H2···O^i^	0.848 (17)	2.292 (17)	2.9955 (15)	140.6 (14)
N2—H2···O^ii^	0.848 (17)	2.434 (16)	3.1333 (15)	140.3 (14)
O—H0···S1^iii^	0.857 (19)	2.299 (19)	3.1349 (10)	165.2 (16)

Symmetry codes: (i) −*x*+1, −*y*+1, −*z*+1; (ii) *x*−1, *y*−1, *z*−1; (iii) *x*+1, *y*+1, *z*+1.
